# Altered neurovascular coupling in children with idiopathic generalized epilepsy

**DOI:** 10.1111/cns.14039

**Published:** 2022-12-08

**Authors:** Jie Hu, Haifeng Ran, Guiqin Chen, Yulun He, Qinghui Li, Junwei Liu, Fangling Li, Heng Liu, Tijiang Zhang

**Affiliations:** ^1^ Department of Radiology The Affiliated Hospital of Zunyi Medical University, Medical Imaging Center of Guizhou Province Zunyi China; ^2^ Department of Radiology and Nuclear Medicine Xuanwu Hospital, Capital Medical University Beijing China

**Keywords:** children, functional magnetic resonance imaging, idiopathic generalized epilepsy, neurovascular coupling

## Abstract

**Aims:**

Alterations in neuronal activity and cerebral hemodynamics have been reported in idiopathic generalized epilepsy (IGE) patients, possibly resulting in neurovascular decoupling; however, no neuroimaging evidence confirmed this disruption. This study aimed to investigate the possible presence of neurovascular decoupling and its clinical implications in childhood IGE using resting‐state fMRI and arterial spin labeling imaging.

**Methods:**

IGE patients and healthy participants underwent resting‐state fMRI and arterial spin labeling imaging to calculate degree centrality (DC) and cerebral blood flow (CBF), respectively. Across‐voxel CBF‐DC correlations were analyzed to evaluate the neurovascular coupling within the whole gray matter, and the regional coupling of brain region was assessed with the CBF/DC ratio.

**Results:**

The study included 26 children with IGE and 35 sex‐ and age‐matched healthy controls (HCs). Compared with the HCs, the IGE group presented lower across‐voxel CBF‐DC correlations, higher CBF/DC ratio in the right posterior cingulate cortex/precuneus, middle frontal gyrus, and medial frontal gyrus (MFG), and lower ratio in the left inferior frontal gyrus. The increased CBF/DC ratio in the right MFG was correlated with lower performance intelligence quotient scores in the IGE group.

**Conclusion:**

Children with IGE present altered neurovascular coupling, associated with lower performance intelligence quotient scores. The study shed a new insight into the pathophysiology of epilepsy and provided potential imaging biomarkers of cognitive performances in children with IGE.

## Graphical abstract

Altered neuronal activity and cerebral vascular hemodynamics have been confirmed in idiopathic generalized epilepsy (IGE) patients, thus, the coupling between neuronal activity and cerebral blood flow may be impaired, but neuroimaging evidence on neurovascular decoupling remains scarce. Here, Jie Hu et al confirmed the impairment of neurovascular coupling (NVC) in IGE children by neuroimaging.

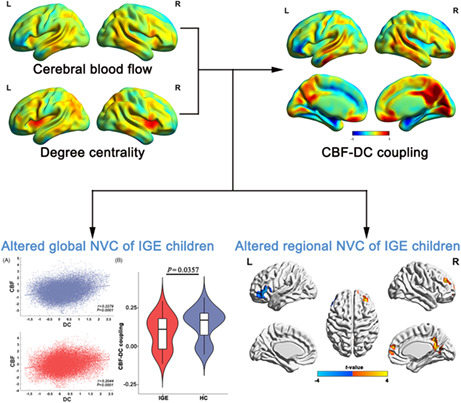

## 1 INTRODUCTION

Epilepsy is one of the most common central nervous system diseases, affecting an estimated 70 million patients worldwide; it causes a significant psychological and economic burden to patients, their families, and society.^1^, ^2^, ^3^ In particular, pediatric epilepsy patients account for approximately 10.5 million cases,^3^, ^4^ and have different clinical manifestations and treatment modalities from adults. Importantly, the frequent seizures may affect the cognitive performance and physical growth of these children, possibly resulting in low intelligence.^5^ Thus, early diagnosis and identification of novel imaging biomarkers of cognitive impairment are critical to improve the prognosis of children with epilepsy.

Idiopathic generalized epilepsy (IGE) is a common subtype of pediatric epilepsy; early diagnosis of IGE is challenging because the etiology is unknown and no sensitive and specific biomarkers have been identified. Magnetic resonance imaging (MRI) is a reliable and noninvasive technique to study the pathological mechanisms of epilepsy in the human brain, analyzing the brain structure and function in an omnidirectional and multangular manner.^6^ Seizure activity in IGE patients may affect the cerebral blood flow (CBF) and metabolism, and neuroimaging evidence of neuronal injury and cerebral perfusion changes in IGE has been provided by resting‐state functional MRI (rs‐fMRI) studies.^7^, ^8^, ^9^, ^10^, ^11^ However, these studies on IGE mainly used a single imaging modality, and this method cannot comprehensively and sensitively reflect the altered regional CBF and neuronal activity caused by seizures.^10^, ^12^ In contrast, combining rs‐fMRI and arterial spin labeling (ASL) imaging can promptly and comprehensively detect altered neurovascular coupling (NVC), reflecting the relationship between changes in neural activity and in regional CBF.^13^, ^14^, ^15^ NVC is a mechanism of the neurovascular unit (NVU) that regulates CBF to meet the energy demands of the neuronal activity and maintain balance.^16^ The metabolic demand increases during seizures, and the CBF has an insufficient response to meet this demand.^17^ These alterations ultimately result in impaired NVC in epilepsy patients. Several studies have identified NVC alterations in schizophrenia,^18^ neuromyelitis optica,^19^ primary open‐angle glaucoma,^20^ and type 2 diabetes,^21^ indicating a possible neuropathological mechanism causing brain dysfunction. These studies have shown that investigating NVC by multimodal MRI may provide novel insights on the pathophysiology of neurological diseases.^18^, ^19^, ^20^, ^21^

Furthermore, basic research has confirmed that the blood–brain barrier is damaged in mammals with seizure.^22^ Recurrent epileptic seizures result in neuronal injury, blood–brain barrier impairment, and gliosis, which alter the integrity of the NVU and eventually may lead to NVC impairment.^22^, ^23^, ^24^, ^25^

However, to the best of our knowledge, specific imaging biomarkers and neuroimaging evidence of NVC alterations in children with IGE are still lacking. Thus, the current study aimed to evaluate the neurovascular decoupling and its clinical significance in childhood IGE combining rs‐fMRI and ASL and provide a new perspective to understand the neuropathological mechanisms of this disease.

## 2 METHODS

### 2.1 Study design and participants

Ethics approval of this research was granted by the Ethic Committee of the Affiliated Hospital of Zunyi Medical University; all steps involving human subjects adhered to the Declaration of Helsinki. Parents or guardians of each participant provided written informed consent to participate in the research.

The children with IGE were recruited in the outpatient department of the Pediatric Neurology Clinic of the Affiliated Hospital of Zunyi Medical University. IGE was diagnosed by two experienced specialists in pediatric neurology based on the International League Against Epilepsy (ILAE) criteria.^26^ The inclusion criteria for the case group were as follows: (1) clinically diagnosed IGE; (2) unremarkable routine head MRI examination; (3) no history of substance abuse; and (4) age 6–16 years at the time of MRI scanning. The exclusion criteria were: (1) any other neurological disorders and/or history of malignant tumors or head trauma; (2) image artifacts affecting the image analysis; and (3) head motion with maximum displacement >2 mm or >2° rotation noted during rs‐fMRI data preprocessing.

For the control group, the inclusion criteria were (1) age 6–16 years at the time of MRI scanning; (2) no neurologic, psychiatric, or systemic illnesses; and (3) no contraindications to MRI. The exclusion criteria were (1) any unusual findings in conventional brain MR images; (2) head motion with maximum displacement >2 mm or >2° rotation visible during rs‐fMRI data preprocessing.

### 2.2 Neuropsychological assessment

On the same day of the MRI scan, all IGE children underwent standardized neuropsychological assessments, performed by an experienced neuropsychologist. The Wechsler Intelligence Scale for Chinese Children Revised Version is an individually administered instrument for assessing the cognitive performance of children between the ages of 6 and 16 years, widely used in China.^27^, ^28^ Neuropsychological assessments were conducted in a quiet illuminated room, with only the participant and neuropsychologist present. This assessment can measure three intelligence quotient (IQ) variables in each subject.

### 2.3 Acquisition of MRI data

After the neuropsychological assessment, the children were allowed a rest period before the imaging scans. Then, all participants underwent MRI scans using a 3.0‐T magnetic resonance scanner (GE Healthcare, Chicago, IL, USA). A three‐dimensional brain volume sequence (3D BRAVO) was applied to obtain structural T1‐weighted images (repetition time [TR]: 1900 ms; echo time [TE]: 2.1 ms; inversion time [TI]: 900 ms; flip angle: 9°; slice thickness: 1.0 mm; matrix: 256 × 256). The functional images were acquired using a gradient‐echo echo‐planar imaging sequence (TR: 2000 ms; TE: 30 ms; voxel size: 3.75 mm × 3.75 mm × 4 mm; FOV: 240 mm × 240 mm; slice number: 33; slice thickness: 4 mm; flip angle: 90°). The perfusion images were obtained with a pseudo‐continuous ASL sequence (TR: 4599 ms; TE: 9.8 ms; slice number: 36; FOV: 256 mm × 256 mm; slice thickness: 4 mm; flip angle: 90°). Before each scan, every subject was instructed to relax, close their eyes while staying awake, and keep still.

### 2.4 Rs‐fMRI data preprocessing

For all the collected rs‐fMRI data, preprocessing was performed with DPABI V5.0 ^29^(http://rfmri.org/DPABI), implemented in MATLAB R2018b (MathWorks, Inc., Natick, MA, USA; https://mathworks.com). The DICOM images were converted into the Nifti format. The first 10 volumes were eliminated to stabilize the signal of the images, and slice timing and realignment was performed on the remaining 200 volumes. The head motion was corrected with the Friston 24‐parameter model. Linear drift corrections were applied, and the interference of white matter, cerebrospinal fluid, and movement signals was removed using a regression. Then, the functional images were spatially normalized using a standard template in the Montreal Neurological Institute (MNI) space. The frequency range (0.01–0.1 Hz) was retained using a temporal bandpass filter. Finally, the functional images were re‐sampled into 3‐mm cubic voxels for further analysis.

### 2.5 CBF data preprocessing

The CBF maps were obtained from the ASL sequence, and the SPM12 toolbox (http://www.fil.ion.ucl.ac.uk/spm) in MATLAB was chosen for the preprocessing of these images. First, the CBF map of each participant was spatially normalized into the MNI space using the one‐step registration method, with voxel resampling to 3 × 3 × 3 mm. Then, further transformations were performed to standardize the maps, converting the CBF map of each participant into z‐scores with the following method: within the whole‐brain mask, the voxel‐wise z‐scores were calculated from each voxel's CBF value minus the mean CBF value of all voxels, divided by the standard deviation of all CBF values. Finally, the standardized CBF maps were smoothed using a 6‐mm full‐width at half‐maximum (FWHM) kernel.

### 2.6 Calculation of degree centrality

Degree centrality (DC) represents the number of edge connections between a voxel and all the other voxels in the brain.^30^ In the present study, Pearson's correlation coefficients were calculated using a BOLD time series between pairs of voxels within the whole‐brain mask, removing the global signal. Given the ambiguous interpretation of the negative correlations to remove weak correlations after removing the global signal, we conservatively restricted the DC analyses to positive correlations with *r* ≥ 0.2. The DC calculations were performed using DPARSFA (http://rfmri.org/DPARSF). The DC maps were then standardized into z‐scores, and smoothed with a 6‐mm FWHM kernel.

### 2.7 Across‐voxel CBF‐DC coupling analysis

In each participant, across‐voxel correlation analyses were performed between DC and CBF in the whole gray matter (GM) to quantitatively evaluate the global NVC. The Pearson correlation coefficients between the CBF and DC values of all voxels within the GM mask were calculated for each participant. The coefficients from the two groups were tested for normality and variance homogeneity. Finally, two‐tailed, unpaired two‐sample t‐tests were used to compare the HC and IGE groups if the values were normally distributed, and Mann–Whitney tests otherwise. *p* values <0.05 were considered statistically significant.

### 2.8 Voxel‐Wise analysis of CBF/DC ratio, CBF, and DC

For all voxels, we calculated the CBF/DC ratio using the original values of CBF and DC, without z‐transformations, to represent the regional NVC. For each subject, the CBF/DC ratio map was then standardized into z‐scores, and smoothed using a 6‐mm FWHM kernel. We performed voxel‐wise comparisons to identify significant intergroup differences in the CBF/DC ratio. Intergroup differences in CBF and DC were also analyzed to determine the causes of the differences in the CBF/DC ratio.

### 2.9 Statistical analysis

#### 2.9.1 Group differences in clinicodemographic variables

The normality of the distribution of age and years of education was assessed with the Kolmogorov–Smirnov test. Normally distributed data were analyzed with the unpaired two‐sample *t*‐test; otherwise, the Mann–Whitney *U*‐test was used. Age and years of education were normally distributed and analyzed with unpaired two‐sample t‐tests. The Chi‐squared test was performed to analyze the intergroup difference in sex distribution. SPSS version 17.0 (IBM, Armonk, NY, USA) was used to perform these statistical analyses, and the significance level was set as *p* < 0.05.

#### 2.9.2 Voxel‐wise intergroup comparation in CBF, DC, and CBF/DC ratio maps

The differences in CBF, DC, and CBF/DC ratio maps were analyzed using a voxel‐wise two‐sample t‐test in the DPABI software V5.0 (http://rfmri.org/DPABI), based on the MATLAB R2018b platform, adjusting for the covariates of age, sex, and years of education. For the resulting CBF, DC, and CBF/DC ratio maps, the Gaussian random field (GRF) method (*p* < 0.05) was selected to correct for multiple comparisons.

#### 2.9.3 Group difference in global NVC and correlation analysis

The CBF‐DC correlation coefficients of the two groups were tested for normality using the Kolmogorov–Smirnov test; the unpaired two‐sample t‐test was used for data with normal distribution, and the Mann–Whitney *U*‐test otherwise. The mean value of each cluster with significant between‐group differences in CBF, DC, and CBF/DC ratio was extracted and correlated with the clinical variables using Pearson correlation. SPSS (Version 17.0) was used to perform these statistical analyses, and the significance level was set as *p* < 0.05.

#### 2.9.4 Validation analysis

We validated our results considering four potential influencing factors: gray matter volume (GMV) changes, demographic factors (sex, age, and years of education), different DC correlation thresholds (i.e., 0.15 and 0.25), and the influence of antiepileptic drugs.^30^, ^31^, ^32^, ^33^ The analyses were repeated in all the resulting statistical CBF, DC, and CBF/DC ratio maps and corrected using the GRF method (*p* < 0.05).

In addition, we replaced the DC with fractional amplitude of low frequency fluctuations (fALFF) to assess the neuronal activity. All preprocessing steps were performed using the same parameters as in the DC preprocessing. Then, the voxel‐wise CBF/fALFF ratio was calculated and compared between the two groups, controlling for the effects of sex, age, and years of education. The GRF method (*p* < 0.05) was used to correct for multiple comparisons. We have also performed the spatial normalization for all children by using the pediatric brain template^34^ to validate our results. And the processing workflow of replacing the pediatric brain template has been added to the Supplementary Materials.

## 3 RESULTS

### 3.1 Participants

The study finally included 61 right‐handed subjects (26 children with IGE and 35 healthy controls [HCs]), some of whom were also included in our previous studies.^11^, ^35^ Among the children with IGE, 16 patients were regularly administered antiepileptic drugs in accordance with the ILAE treatment guidelines. Eight participants were using valproate sodium, four a combination of valproate sodium and oxcarbazepine, two valproate sodium and clonazepam, one valproate sodium and levetiracetam, and one oxcarbazepine. The remaining 10 children with IGE were not on antiepileptic drugs at the time of scanning. The groups were not significantly different in sex, age, or education years. The clinical and demographic characteristics of the participants are summarized in Table 1.

**TABLE 1:** Demographic characteristics of the sample

Characteristics	IGE	HCs	Statistics	*p*
Number of subjects	26	35		
Gender (female/male)	13/13	16/19	*χ* ^*2*^ = 0.110	0.740
Age (years)	11.69 ± 2.99	11.88 ± 2.48	*t* = −0.276	0.784
Education (years)	6.11 ± 2.71	6.85 ± 3.03	*t* = −0.987	0.328
Duration of illness (years)	4.12 ± 3.89	NA	NA	NA
Age of disease onset (years)	7.60 ± 3.92	NA	NA	NA
Verbal intelligence quotient	90.07 ± 20.08	NA	NA	NA
Performance intelligence quotient	84.00 ± 16.76	NA	NA	NA
Full‐scale intelligence quotient	86.53 ± 18.26	NA	NA	NA

*Note*: Data are presented as the mean ± standard deviation.

Abbreviations: HCs, healthy controls; IGE, idiopathic generalized epilepsy; NA, not be acquired.

### 3.2 Spatial distributions of CBF, DC and the CBF/DC ratio

Despite subtle differences, the spatial distributions of CBF, DC, and CBF/DC ratios in the IGE group were similar to those in the HCs group (Figure S1). A higher CBF was primarily noted in the IGE group in the posterior cingulate cortex, parietal cortex, visual cortex, and lateral temporal cortex. In contrast, a lower DC emerged mostly in the posterior cingulate cortex, precuneus, inferior frontal cortex, and lateral temporal and parietal cortex. Higher CBF/DC ratios were identified in the medial prefrontal cortex, inferior temporal gyrus, posterior cingulate cortex, and precuneus.

### 3.3 Changes in whole gray matter CBF‐DC coupling

**FIGURE 1:**
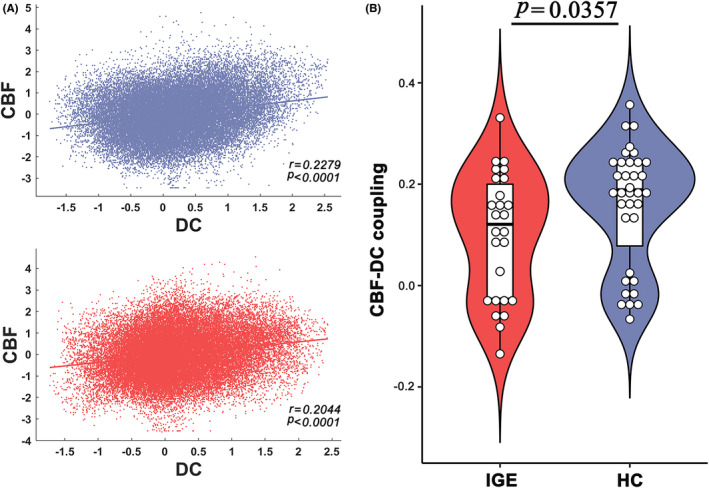
CBF‐DC coupling changes in children with IGE. (A) Across‐voxel spatial correlation between CBF and DC in two participants (violet: HC, *r* = 0.2279; red: IGE, *r* = 0.2044). (B) CBF‐DC coupling within the GM mask was significantly lower in the IGE group than in the HCs (*p* < 0.05). CBF: cerebral blood flow; DC: degree centrality; HCs: healthy controls; IGE: idiopathic generalized epilepsy.

All subjects exhibited significant across‐voxel spatial correlations between CBF and DC; two representative correlation maps (one from each group) are presented in Figure 1A. At the group level, global CBF and DC were slightly lower in the IGE group (CBF: 53.42 ± 8.37 ml/100 g/min; DC: 0.011 ± 0.010) than in the HC group (CBF: 53.85 ± 7.60 ml/100 g/min; DC: 0.016 ± 0.008; two‐sample *t*‐test, CBF: *t* = −0.208, *p* = 0.836; DC: *t* = −1.705, *p* = 0.093). A significant correlation was found between CBF and DC in both groups; however, the CBF‐DC coupling within the GM mask was significantly lower in the IGE group than in the HCs group (*p* < 0.05, Mann–Whitney *U*‐test; Figure 1B).

### 3.4 Changes in CBF, DC, and CBF/DC ratio

**FIGURE 2:**
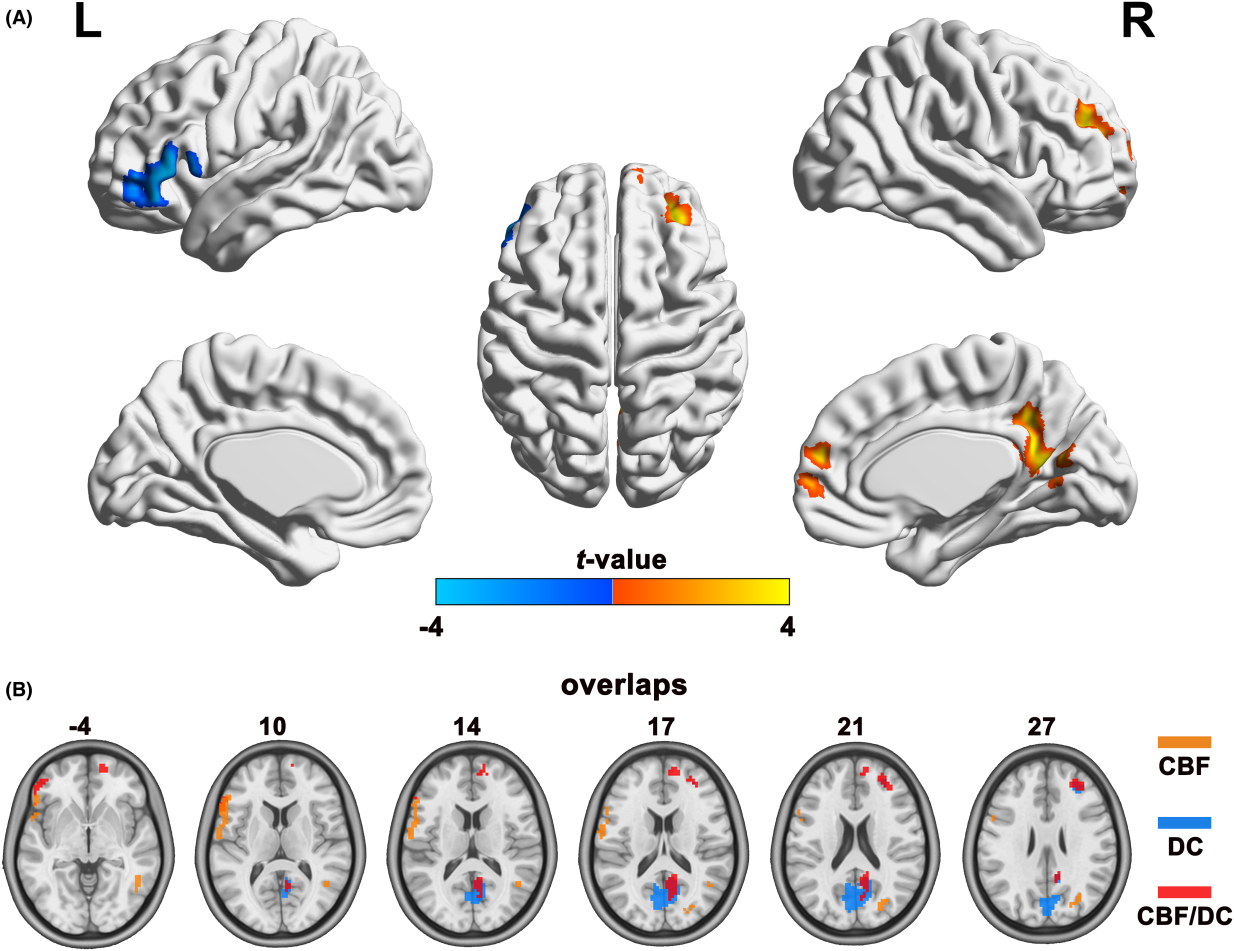
Comparison of the alterations in CBF/DC ratio between the IGE and HCs groups, controlling for the influence of age, sex, and years of education (GRF corrected: *p* < 0.05). (A) The cold and warm colors represent the areas with significantly decreased and increased CBF/DC ratio in IGE children, respectively. (B) Overlay of clusters with significant between‐group differences in CBF (orange), DC (blue), and CBF/DC ratio (red). CBF: cerebral blood flow; DC: Degree centrality; HCs: healthy controls; L: left; R: right; IGE: idiopathic generalized epilepsy.

The IGE group showed significantly higher CBF/DC ratio in the right medial frontal gyrus (MFG), posterior cingulate cortex/precuneus, and middle frontal gyrus, with significantly lower CBF/DC ratio in the left inferior frontal gyrus compared to the HCs group (GRF corrected: *p* < 0.05); all results are shown in Table 2 and Figure 2A. The IGE group also showed a significantly lower CBF than the HCs group in the left inferior frontal gyrus, and a higher CBF in the right middle temporal gyrus and superior parietal lobule (GRF corrected: *p* < 0.05, Table 2). The IGE group exhibited a lower DC in the right posterior cingulate cortex, precuneus, and middle frontal gyrus compared to the HCs group, though the brain region of higher DC was not reported (GRF corrected: *p* < 0.05, Table 2). We projected the intergroup difference maps of CBF, DC, and CBF/DC ratios onto an overlay map to represent more intuitively the cause of the changes in the CBF/DC ratio (Figure 2B).

**TABLE 2:** Brain regions with significant intergroup differences in CBF, DC, and CBF/DC ratios

	Voxels, *n*	MNI coordinates, mm (x, y, z)			Brain regions	Brodmann area	Peak *t* values
CBF	206	−54	14	1	Left inferior frontal gyrus	45	−4.83
	57	48	52	−2	Right middle temporal gyrus	39	3.73
	127	30	−64	46	Right superior parietal lobule	7	4.38
DC	387	9	−57	18	Right posterior cingulate	31	−4.77
	167	27	24	51	Right middle frontal gyrus	8	−4.37
CBF/DC ratio	67	15	57	−3	Right medial frontal gyrus	10	4.46
	123	−57	24	0	Left inferior frontal gyrus	45	−4.16
	120	9	−54	58	Right posterior cingulate	31	5.02
	71	27	45	21	Right middle frontal gyrus	10	4.30

Abbreviations: CBF, cerebral blood flow; DC, Degree centrality; MNI, montreal neurological institute.

### 3.5 Clinical correlation analysis

**FIGURE 3:**
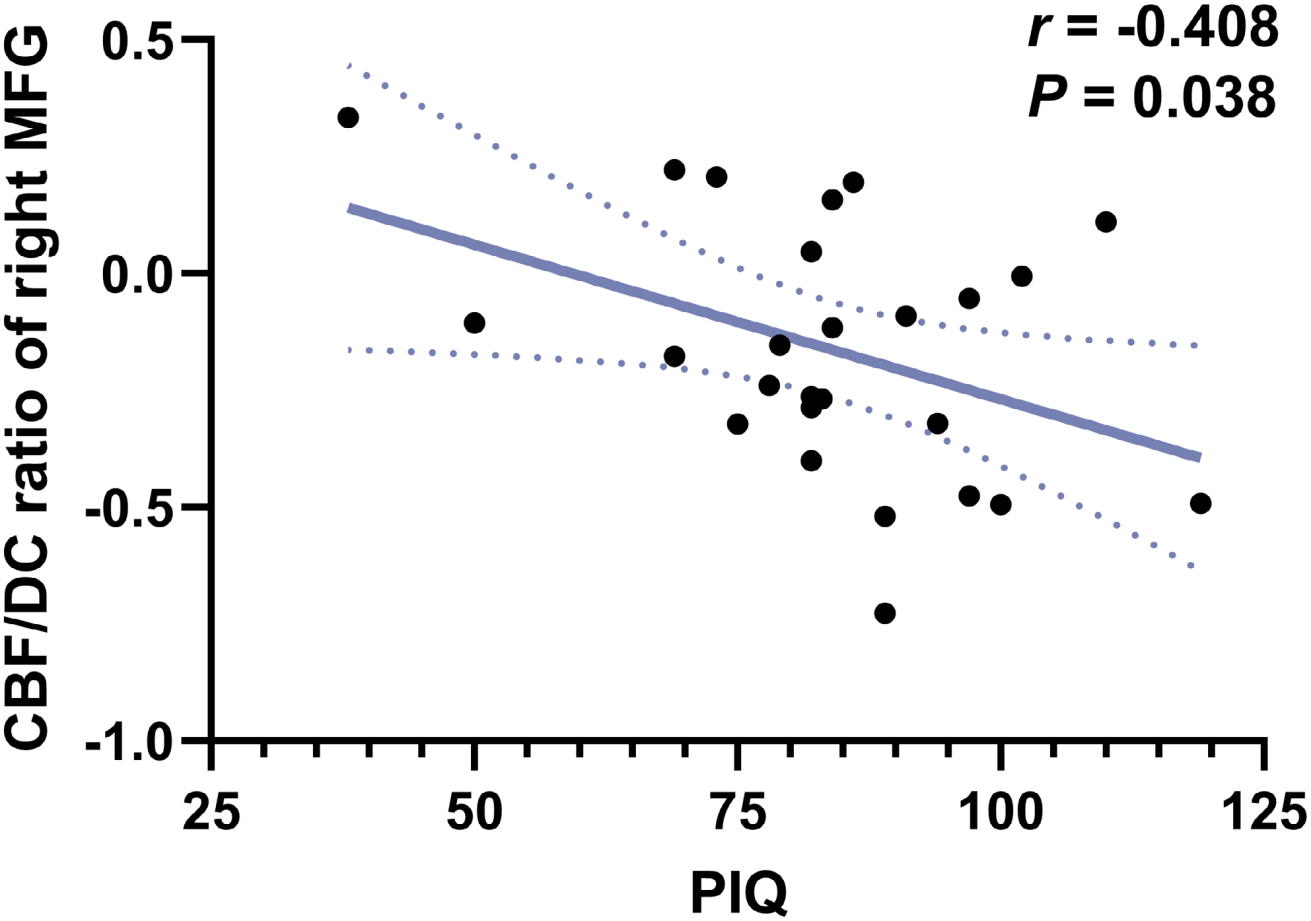
Pearson's correlation between CBF/DC ratios in the right MFG and PIQ scores in IGE patients. CBF: cerebral blood flow; DC: degree centrality; MFG: medial frontal gyrus; PIQ: performance intelligence quotient.

Pearson's correlation was calculated to examine the relationship between the mean CBF, DC, and CBF/DC ratio in every significantly different cluster and IQ scores, age of onset, and disease duration in children with IGE (Table S1). The results showed a significant negative correlation (*r* = −0.408, *p* = 0.038) between the increased CBF/DC ratio in the right MFG and the performance IQ (PIQ) scores (Figure 3). Other parameters, including the mean value of significantly different CBF and DC clusters, did not show significant correlations with IQ scores, age of onset, and disease duration.

### 3.6 Validation analyses

Overall, the alterations of the CBF/DC ratio observed using different validation strategies remained highly consistent with our main findings, after controlling for the GMV changes, demographic factors (sex, age, and years of education), different DC correlation thresholds (i.e., 0.15 and 0.25), and the influence of antiepileptic drugs. The altered CBF/DC ratio was limited to the right MFG, posterior cingulate cortex/precuneus, middle frontal gyrus, and left inferior frontal gyrus (Figures S2, S7–S9, S12–S14). Furthermore, the significantly different CBF and DC clusters had consistent results using different validation strategies (Figures S3–S6, S8–S11).

The analysis was repeated using fALFF in place of DC to assess the neuronal activity and validate the reproducibility of our results. No significant difference between groups emerged in the global CBF‐fALFF coupling (*t* = −0.6739, *p* = 0.503); however, in the regional NVC analysis, the brain regions with significantly altered CBF/fALFF ratio (GRF corrected: *p* < 0.05, Figure S15) were similar to those with altered CBF/DC ratio (GRF corrected: *p* < 0.05). The alterations of the CBF/DC ratio observed by using the pediatric brain template for spatial normalization remained consistent with our main findings, after controlling for sex, age, and years of education (GRF corrected: *p* < 0.05, Figure S16).

## 4 DISCUSSION

NVC impairment is a significant pathophysiological mechanism in the initiation and development of epilepsy.^22^ Our study confirmed the presence of NVC alterations in IGE children, using neuroimaging methods. We found significantly lower global NVC in the GM of IGE children compared to that of HCs. In addition, a voxel‐wise analysis of the CBF/DC ratio in the whole GM demonstrated regional NVC changes in multiple functional regions associated with executive function and cognitive control, undetectable using CBF or DC alone. Another important finding was the increased regional NVC in the right MFG of childhood IGE patients, associated with the executive function. These findings could help improve the understanding of the neuropathological mechanisms of childhood IGE from the new perspective of NVC and provide potential neuroimaging biomarkers for early diagnosis and assessment of cognitive performance in children with IGE.

Previous studies reported significant across‐voxel correlations between CBF and functional connectivity strength, regional homogeneity, and DC in patients with schizophrenia, neuromyelitis optica, and diabetes mellitus.^18^, ^19^, ^21^ These results suggest an important potential role of the NVC in the pathophysiology of the human brain. In our study, an across‐voxel correlation between CBF and DC was found in IGE patients; however, the correlation was significantly lower in the IGE group than in the HCs, suggesting global neurovascular decoupling in IGE. Across‐voxel CBF‐DC correlation may represent a global NVC alteration, possibly resulting from changes in the NVU components.^13^, ^19^ The NVU comprises neurons, astrocytes, and blood vessels, and its structural integrity is critical to retain its function. Impairment of any components can cause NVC alterations.^36^ Neurons are a crucial component of the NVU and potentially the NVC drivers.^37^ GMV studies reported significantly altered GMV in children with IGE^31^, ^38^; this finding may be associated with neuronal damage caused by progressive epileptic seizures and may become an early driver of global NVC alterations. Astrocytes play a key role in adapting the local blood supply to the neuronal activity^39^, ^40^; an astrocyte impairment may affect the information exchange between neurons and vessels, hindering the coordination between neuronal activity and blood flow.^41^ Furthermore, endothelial cells and pericytes have been confirmed to play a significant role in the pathogenesis of epilepsy,^42^, ^43^, ^44^ possibly due to the blood–brain barrier dysfunction caused by alterations of these cells.^22^, ^45^ These factors may also explain the global NVC alteration.

Several potential factors may explain the CBF alterations found in IGE children in the current study. Based on the theory of NVC, CBF changes are controlled by the neuronal activity; therefore, a region with higher neuronal activity increases its blood supply, as shown by the CBF increase.^16^, ^46^ We found that IGE children have significantly higher CBF than HCs in the right middle temporal gyrus and superior parietal lobule and lower CBF in the left inferior frontal gyrus. These regional CBF changes are similar to previous results from rs‐fMRI studies, confirming that the neuronal activity might play an important role in CBF changes.^47^ Another possible explanation of CBF changes in IGE patients may be attributed to neurotransmitter variations, such as GABA or glutamate.^48^, ^49^, ^50^ These molecules play a critical role in regulating the vascular function and transmitting information within the human brain; each neurotransmitter may convey different information to coordinate vasodilation and vasoconstriction. We found that the DC was lower in children with IGE, mainly in the right posterior cingulate cortex/precuneus and middle frontal gyrus, consistent with previous neuroimaging studies.^9^, ^51^ DC changes may contribute to the NVC alterations in these regions. Decreased DC in IGE patients might also be related to structural damage, such as gray^38^, ^52^, ^53^ and white matter alterations.^54^, ^55^ A disrupted structural integrity may result in an impairment of the functional connections.^56^, ^57^

More detailed information about the regional NVC alterations could be obtained using the CBF/DC ratio in children with IGE, in contrast to the across‐voxel CBF‐DC correlation, which only represents a rough approximation of the global NVC alterations. Disruptions in the regional or global NVC may result from a mismatch between the energy demands of the neuronal activity and the blood supply.^58^ In several brain regions, the voxel‐wise analyses indicated significant intergroup differences in the CBF/DC ratio, whereas the differences in CBF and DC were not significant. This result suggests that measuring the CBF/DC ratio may improve the ability to detect significant intergroup differences and identify regional alterations earlier than with a single CBF or DC analysis. In fact, an increased CBF/DC ratio may result from a subtle increase in CBF, decreased DC, or both. However, brain regions with slight changes in CBF or DC will not show significant differences in between‐group comparisons. In contrast, significant between‐group differences in CBF, DC, or both, and not in the CBF/DC ratio, suggest that CBF, DC, and CBF/DC ratio complement each other. Thus, the combined use of these indicators may provide more accurate information to understand the neuropathological mechanisms of childhood IGE. This study found that IGE children have an increased CBF/DC ratio in the right MFG, posterior cingulate cortex, precuneus, and middle frontal gyrus and a decreased CBF/DC ratio in the left inferior frontal gyrus. The NVC alterations in these regions are similar to the findings of previous studies using other methods.^38^, ^59^ The right posterior cingulate cortex/precuneus and middle frontal gyrus showed normal CBF and reduced DC, indicating that the regional NVC disruption in these regions is mainly caused by the DC reduction. In contrast, the left inferior frontal gyrus showed reduced CBF and normal DC. Importantly, in the right MFG the CBF/DC ratio was significantly different between groups, while DC and CBF were not. A previous meta‐analysis reported GMV changes in the MFG,^38^ indicating that the alteration of the CBF/DC ratio may result from GMV changes. However, the results of our validation analyses suggest a change in the CBF/DC ratio independent of GMV changes. Therefore, the CBF/DC ratio may detect early regional changes in IGE patients with slight CBF or DC alterations.

The brain regions with CBF/DC ratio changes are mainly involved in cognitive control and executive function.^60^, ^61^ The MFG, as part of the prefrontal cortex, is important for these functions^62^, ^63^, and microstructural damage may cause executive dysfunction in patients with IGE. The higher CBF/DC ratio in the right MFG was correlated with the executive function scores in the IGE group in the current study. The CBF/DC ratio in the right MFG and the PIQ were negatively correlated. This finding confirms that the MFG is primarily involved in cognitive and executive functions. In addition, the CBF/DC ratio changes in regions mainly involved in cognitive control and executive functions may contribute to an intelligence decline in IGE patients. The middle and inferior frontal gyrus have been reported to be associated with language processing,^64^, ^65^ and the posterior cingulate cortex/precuneus is an important region for concentration and memory.^66^

On the other hand, the CBF/DC ratio, CBF, and DC in all brain regions, except the right MFG, were not significantly associated with the neuropsychological assessment scores, disease duration, or age of onset. Collectively, these results show that altered regional NVC coupling in children with IGE may be responsible for cognitive impairment. Additional research is warranted for a comprehensive understanding of the mechanisms underlying these findings.

This study has some limitations. First, the sample size was relatively small, and the several different subtypes of IGE were not considered; they may be associated with different NVC alterations. Second, we could not completely eliminate the effect of drugs on the NVC, despite treating the medication status as a covariate; the drug dosage could not be systematically analyzed due to the use of different kinds of antiepileptic drugs. Third, the CBF‐DC correlation and CBF/DC ratio are indirect measures of NVC, insufficient to clarify exactly the specific neuropathologic mechanisms underlying the NVC alterations in IGE patients.

## 5 CONCLUSION

Children with IGE present reduced global CBF‐DC coupling, indicating neurovascular decoupling. Furthermore, the regional disrupted CBF‐DC coupling is associated with executive and cognitive dysfunction. These findings provide new neuroimaging evidence of neurovascular decoupling in children with IGE and may be helpful for a deeper understanding of the potential neuropathological mechanisms in seizure generation, providing new biomarkers of cognitive performance in childhood IGE.

## AUTHOR CONTRIBUTIONS

JH and HFR: manuscript writing, study design and data analysis. GQC, YLH and QHL: manuscript revision. JWL and FLL: collection of data or analysis. HL and TJZ: conception, study design and critical review. All authors contributed to the article and approved the submitted version.

## FUNDING INFORMATION

This study was supported by National Natural Science Foundation of China (Grant No. 82171919 and 81960312), Young Outstanding Scientific and technological Talent of Guizhou Province (grant No. Qiankehepingtairencai[2021]5620), and Talent Program for Future Famous Clinical Doctors of Zunyi Medical University (rc220211205).

## CONFLICT OF INTEREST

The authors declare that the study was conducted in the absence of any commercial or financial relationships that could be construed as a potential conflict of interest.

## Supporting information


Appendix S1:

## Data Availability

Data available upon request to the corresponding authors.
