# Supplementary material for: Altered neurovascular coupling in children with idiopathic generalized epilepsy
Source: CNS Neurosci Ther. 2022 Dec 8;29(2):609–18. doi: 10.1111/cns.14039 (PMC9873522; doi:10.1111/cns.14039)
Supplement: Supplementary file 1 — Appendix S1: [file CNS-29-609-s001.docx]

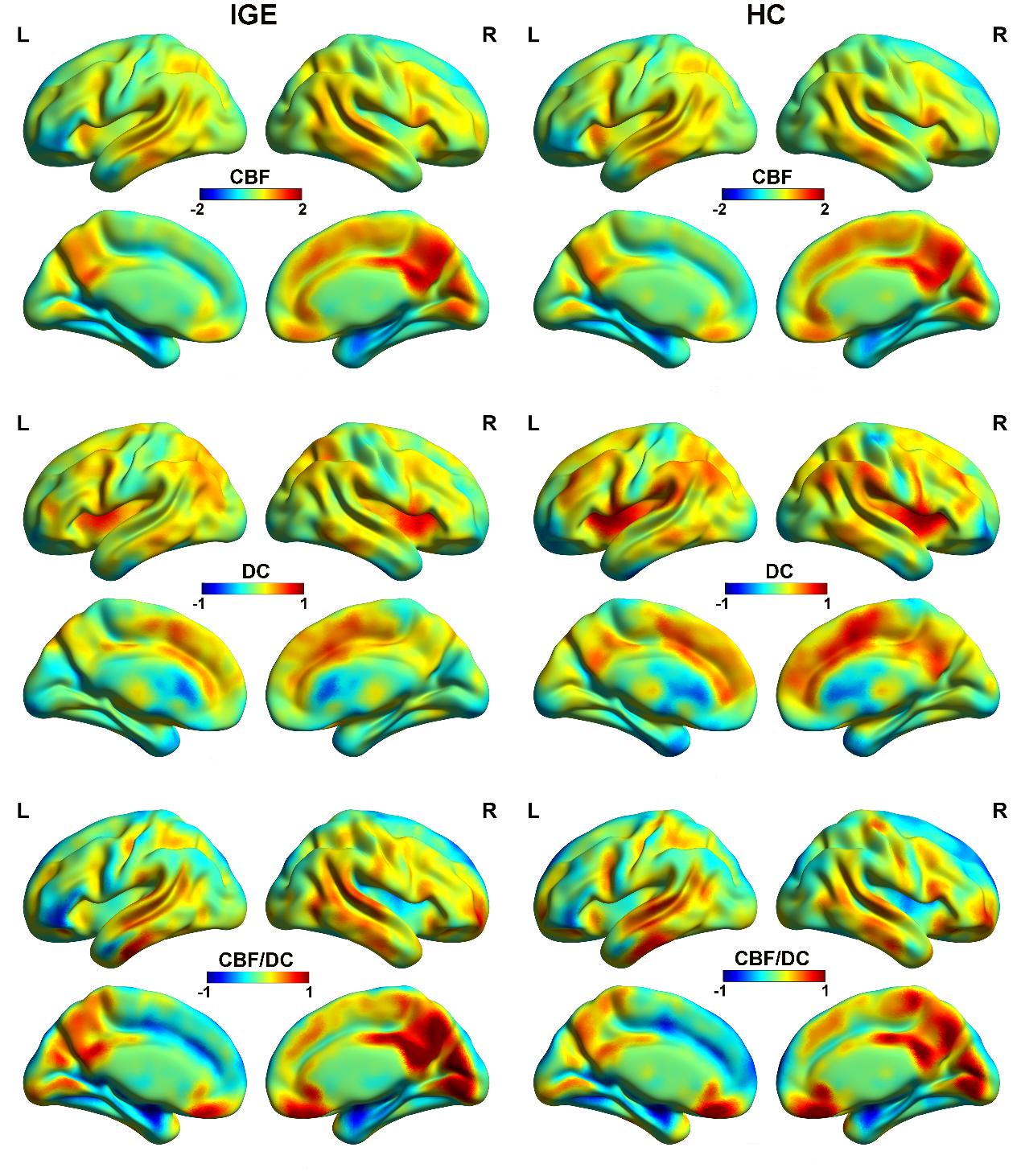


Figure S1 Spatial distribution maps of CBF, DC, and CBF/DC ratio. All Maps were averaged across subjects within groups after normalized to z-scores. A connectivity threshold of 0.2 were selected for DC calculated. Despite subtle differences, the two groups exhibited similar spatial distributions in above measures. HC, healthy controls; L, left; R, right; IGE, idiopathic generalized epilepsy; CBF, cerebral blood flow; DC, degree centrality.


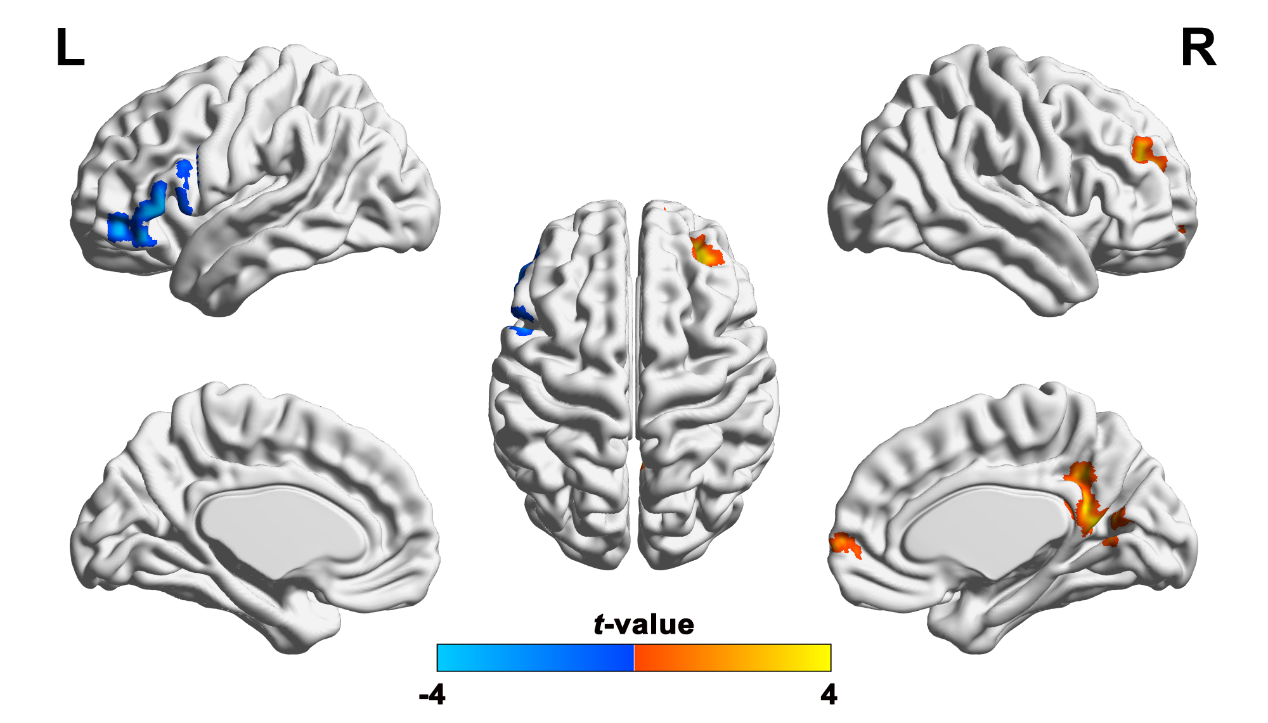


**Figure S2** Comparison of alterations in CBF/DC ratios between the IGE and HC groups after correction for GMV and controlling for the effects of age, sex, and years of education (GRF corrected, P < 0.05). The cold and warm colors represent significantly decreased and increased CBF/DC ratio in the IGE children, respectively.

HC, healthy controls; L, left; R, right; IGE, idiopathic generalized epilepsy; CBF, cerebral blood flow; DC, degree centrality.


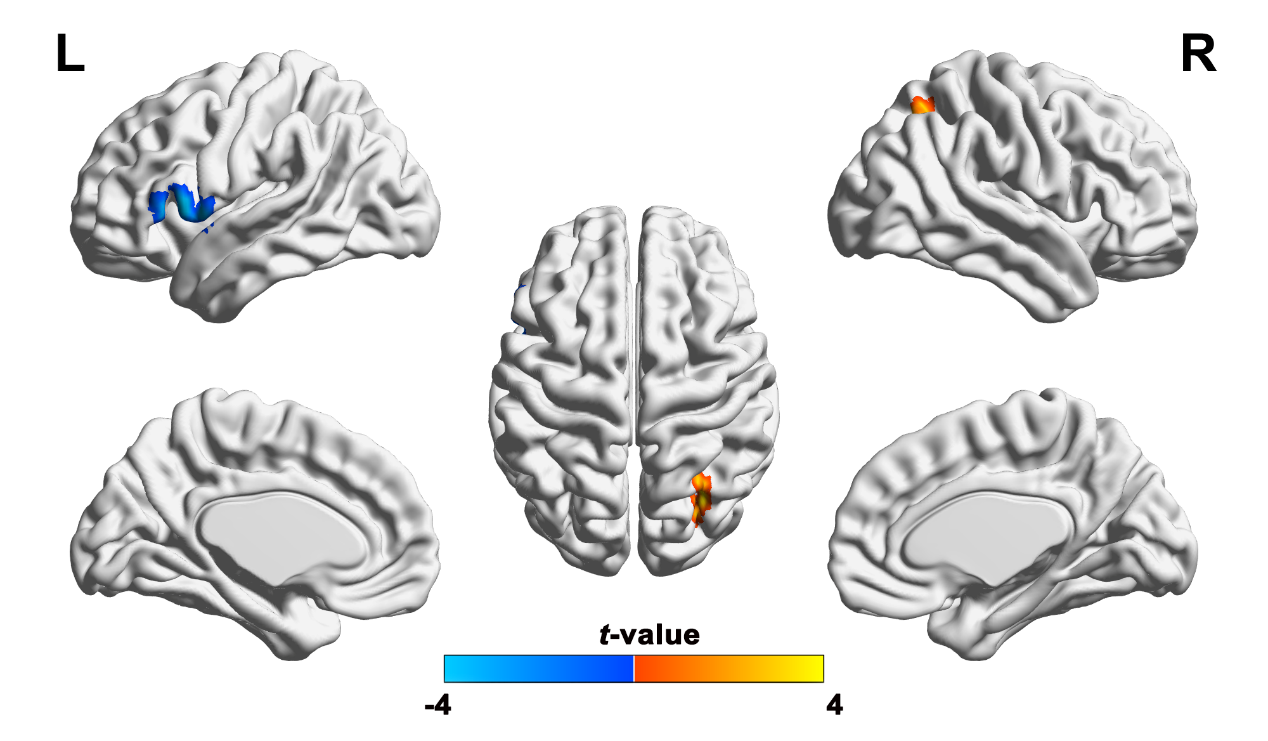
Figure S3 Alterations of CBF in IGE patients compared with HC after correction for GMV and controlling for the effects of age, sex and education (GRF corrected, *P* < 0.05). The warm and cold colors represent significantly increased and decreased CBF in the IGE patients, respectively.

Figure S
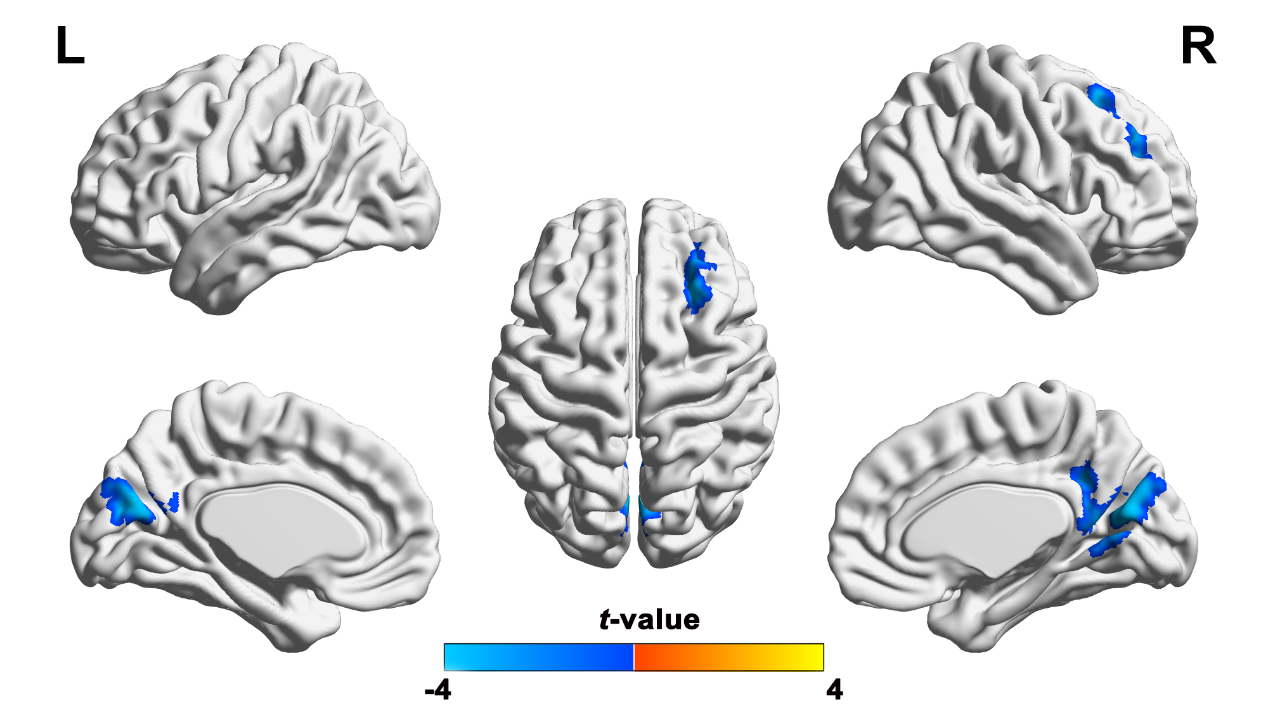
4 Alterations of DC in IGE patients compared with HC after correction for GMV and controlling for the effects of age, sex and education (GRF corrected, *P* < 0.05). The cold color represent significantly decreased DC in the IGE children.


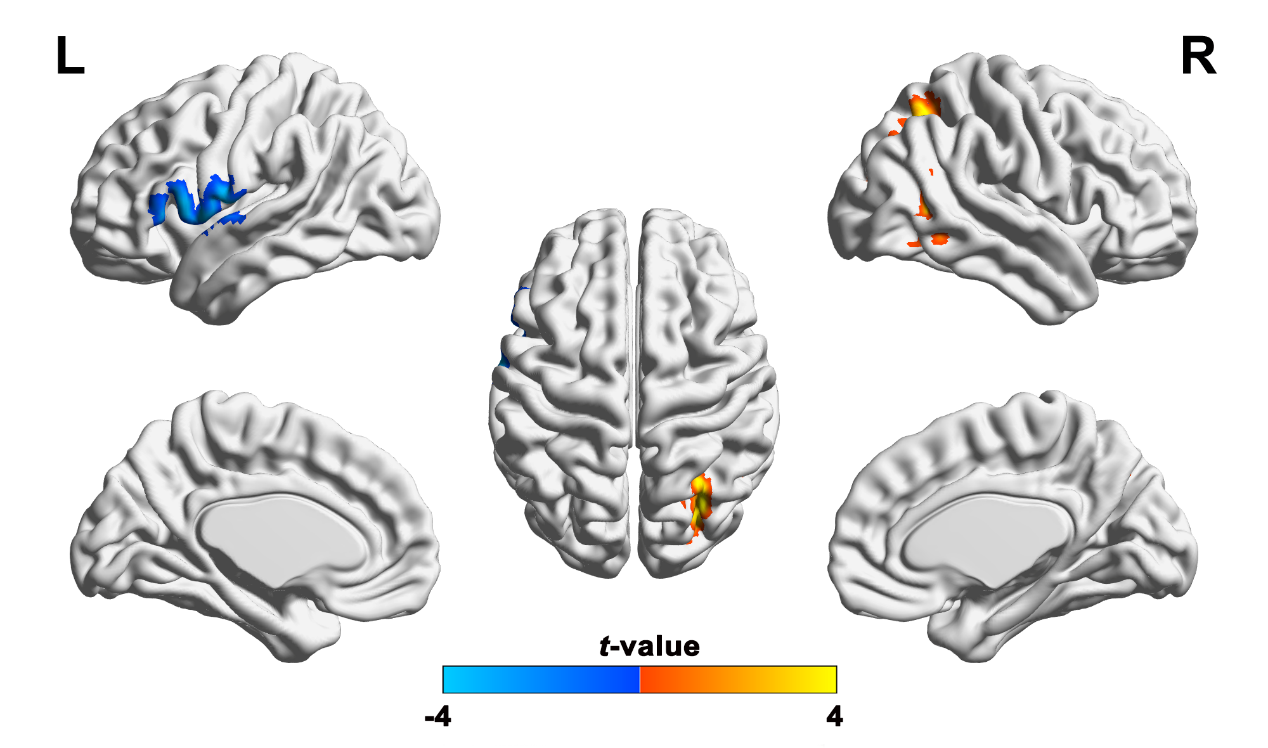
Figure S5 Alterations of CBF in IGE patients compared with HC without controlling for the effects of age, sex and education (GRF corrected, *P* < 0.05). The warm and cold colors represent significantly increased and decreased CBF in the IGE patients, respectively.

Figure S6 Alterations of DC in IGE patients compared with HC without controlling for the effects of age, sex and education (GRF corrected, *P* < 0.05). The warm and cold colors represent significantly increased and decreased DC in the IGE patients, respectively
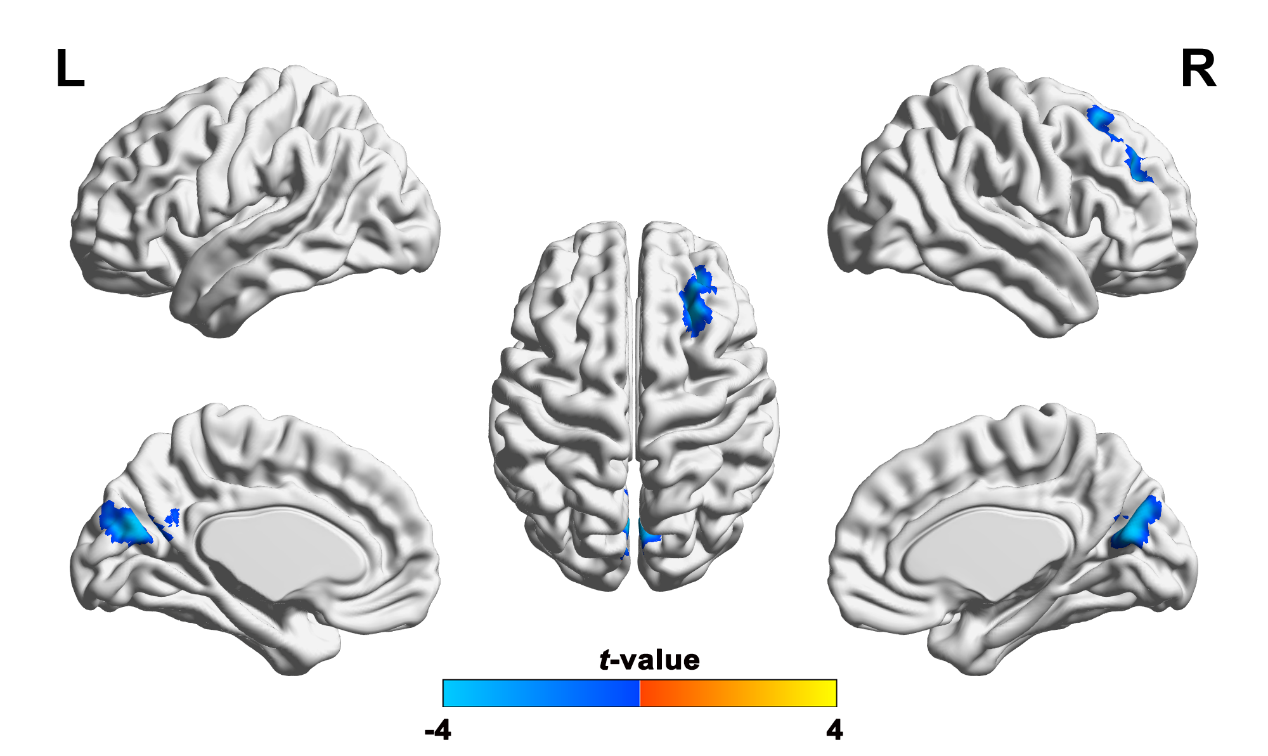
.


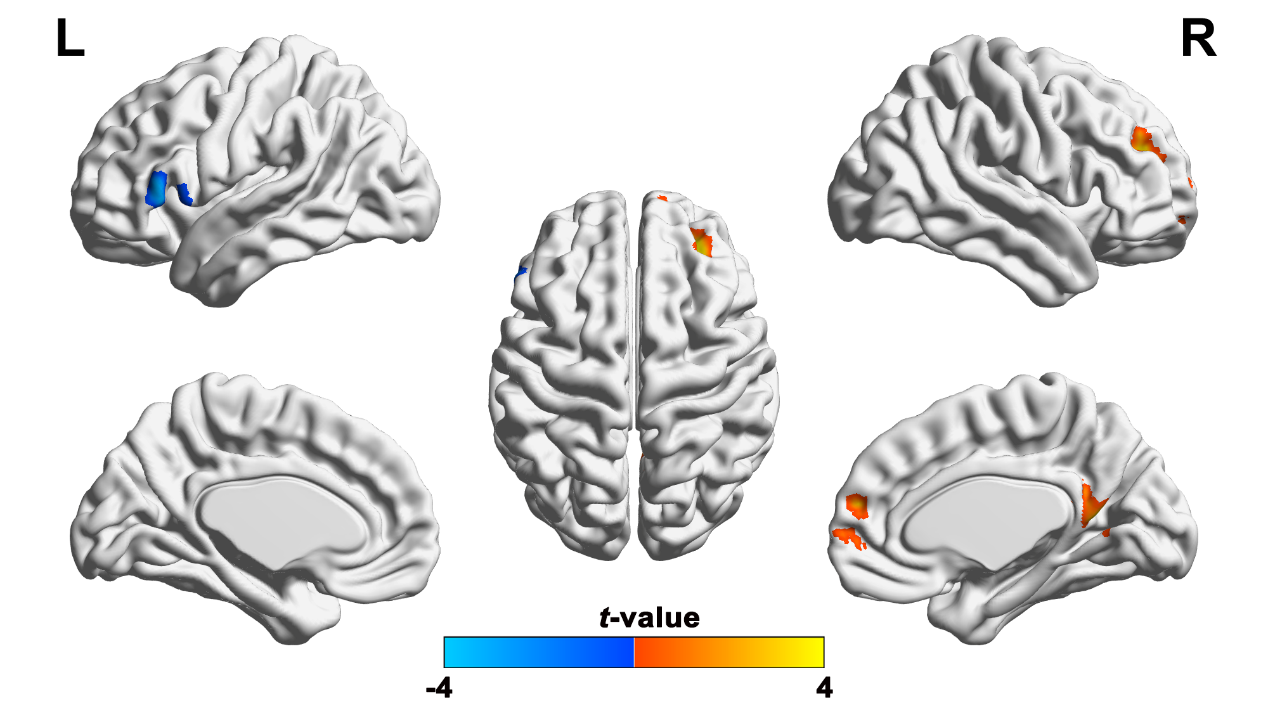
Figure S7 Alterations of CBF/DC ratios in IGE patients compared with HC without controlling for the effects of age, sex and education (GRF corrected, *P* < 0.05). The warm and cold colors represent significantly increased and decreased CBF/DC ratio in the IGE patients, respectively.


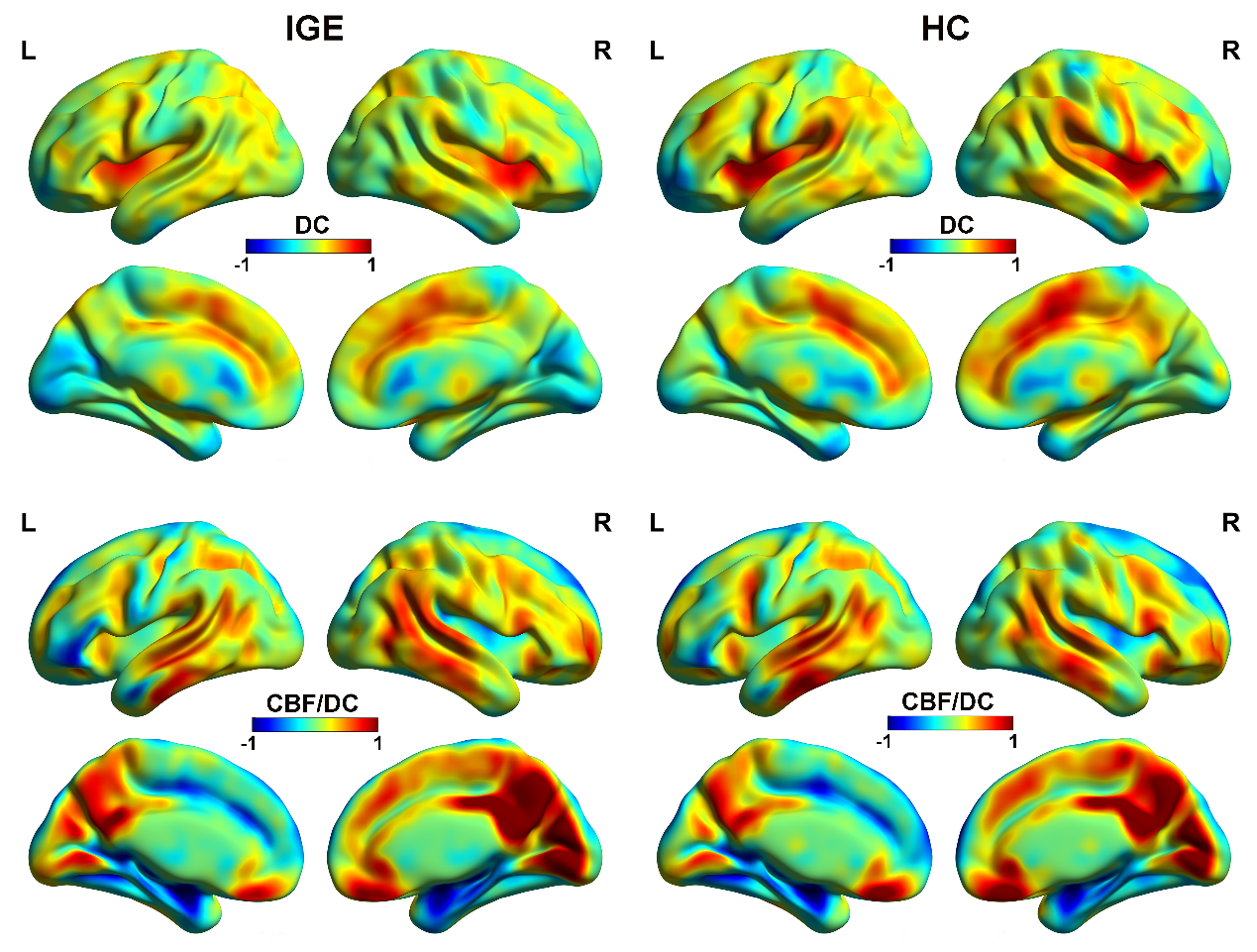
Figure S8 Spatial distribution maps of DC, and CBF/DC ratio. The DC is calculated using a connectivity threshold of 0.15.


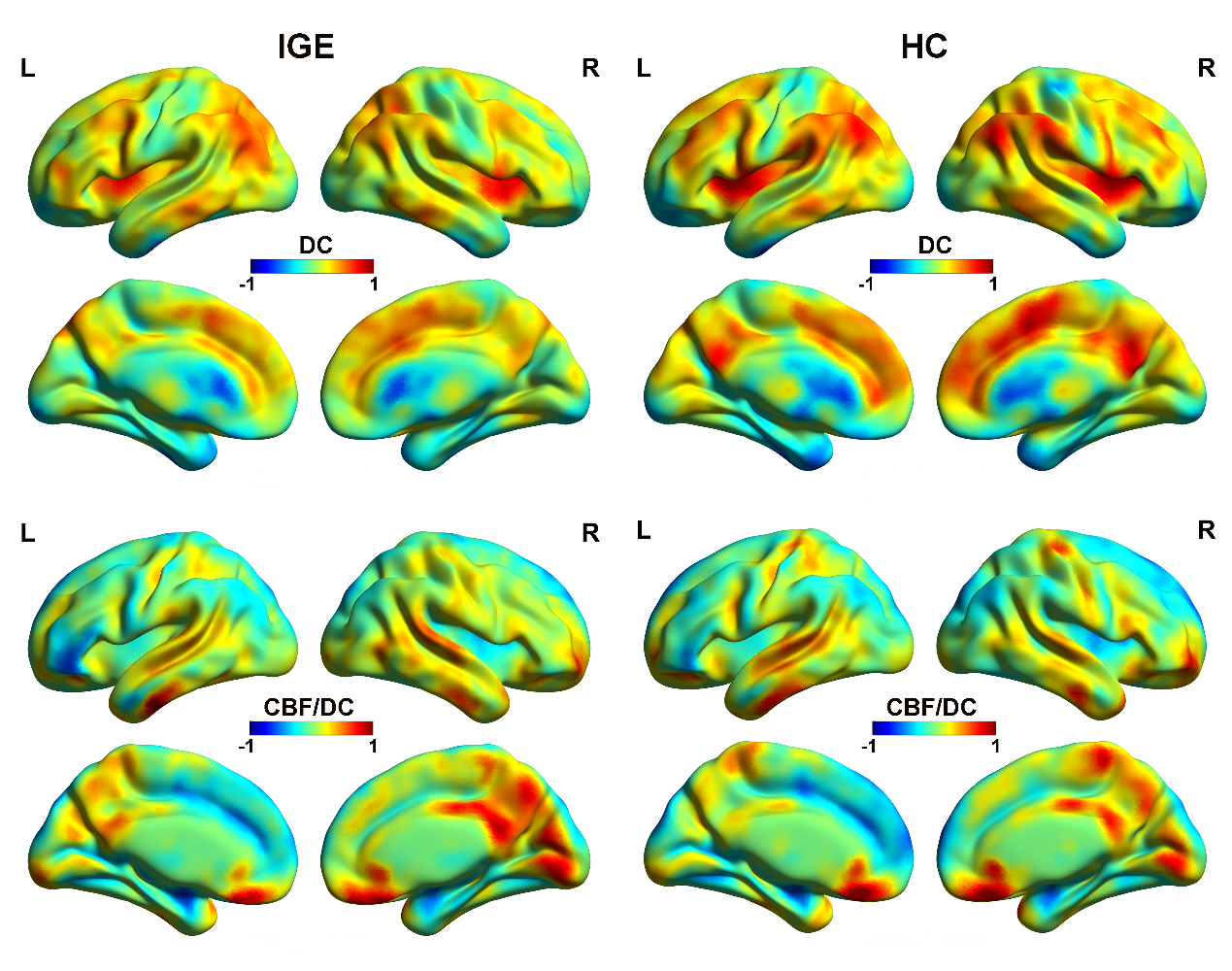
Figure S9 Spatial distribution maps of DC, and CBF/DC ratio. The DC is calculated using a connectivity threshold of 0.25.


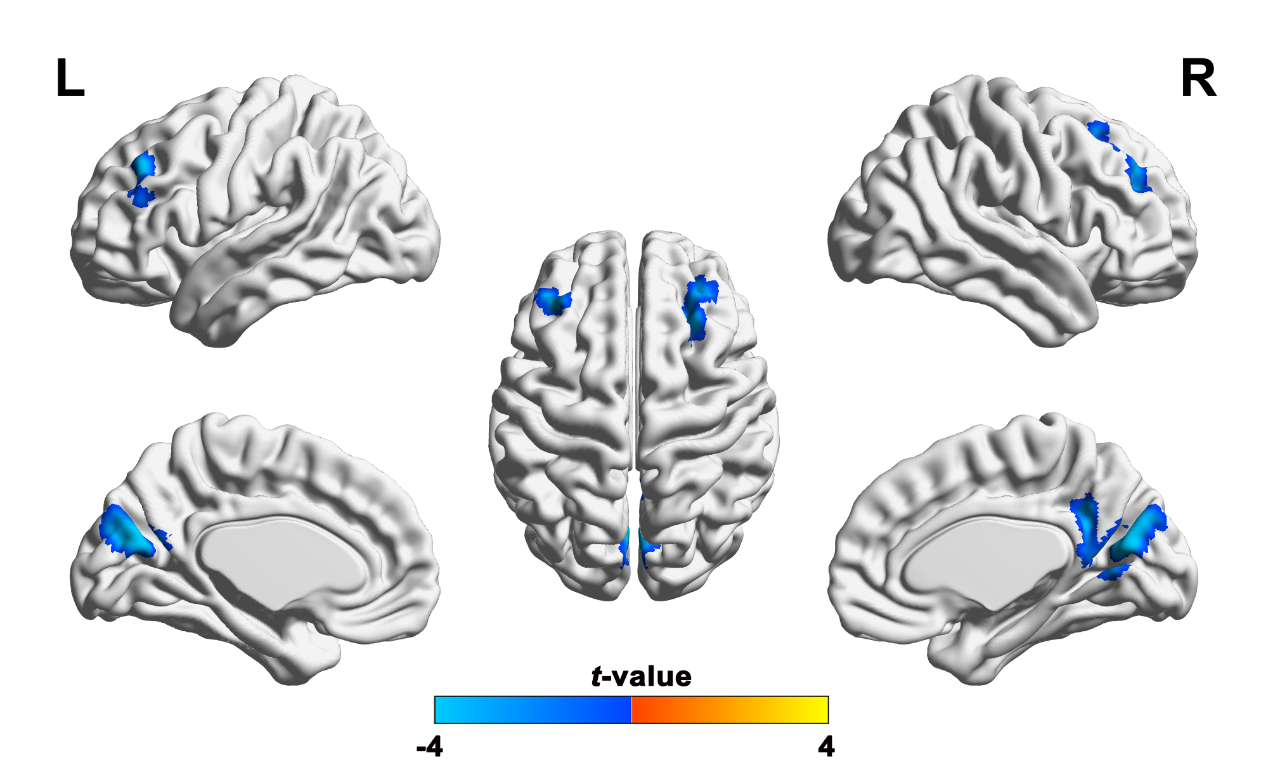
Figure S10 Alterations of DC in IGE patients compared with HC controlling for the effects of age, sex and education (GRF corrected, P < 0.05), while DC is calculated using a connectivity threshold of 0.15. The warm and cold colors represent significantly increased and decreased DC in the IGE patients, respectively.

Figure S11 Alterations of DC in IGE patients compared with HC controlling for the effects of age, sex and education (GRF corrected, P < 0.05), while DC is calculated using a connectivity threshold of 0.25. The warm and cold colors represent significantly increased and decreased DC in the IGE patients, respectively.

Figure S12 Alterations of CBF/DC ratios in IGE patients compared with HC controlling for the effects of age, sex and education (GRF corrected, P < 0.05), while DC is calculated using a connectivity threshold of 0.15. The warm and cold colors represent significantly increased and decreased CBF/DC ratio in the IGE patients, respectively.
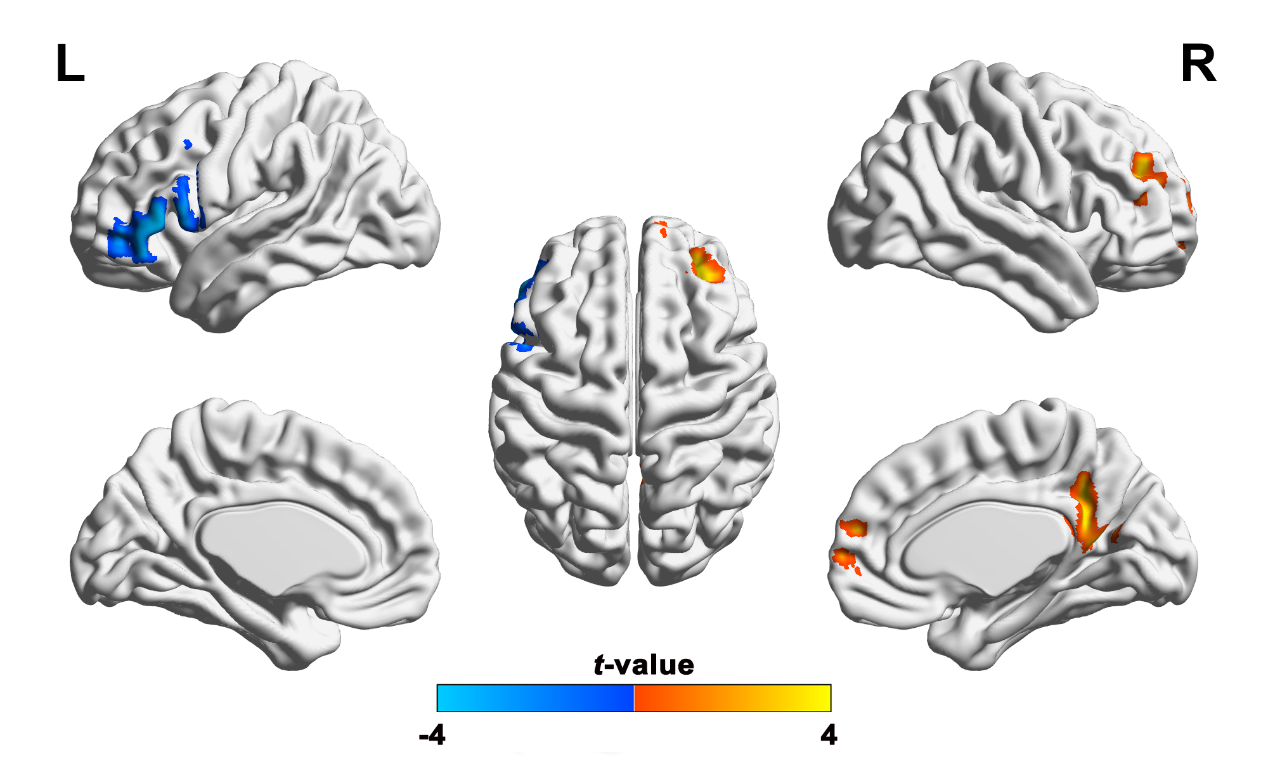

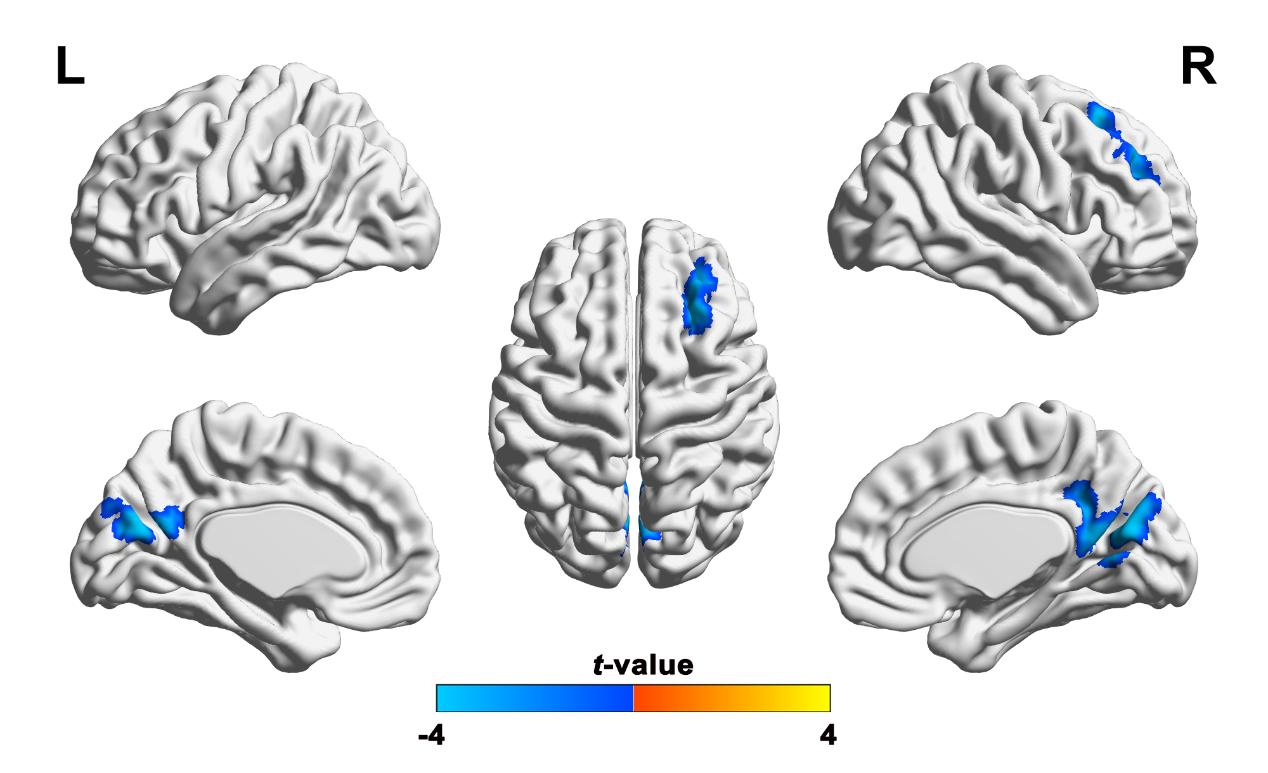


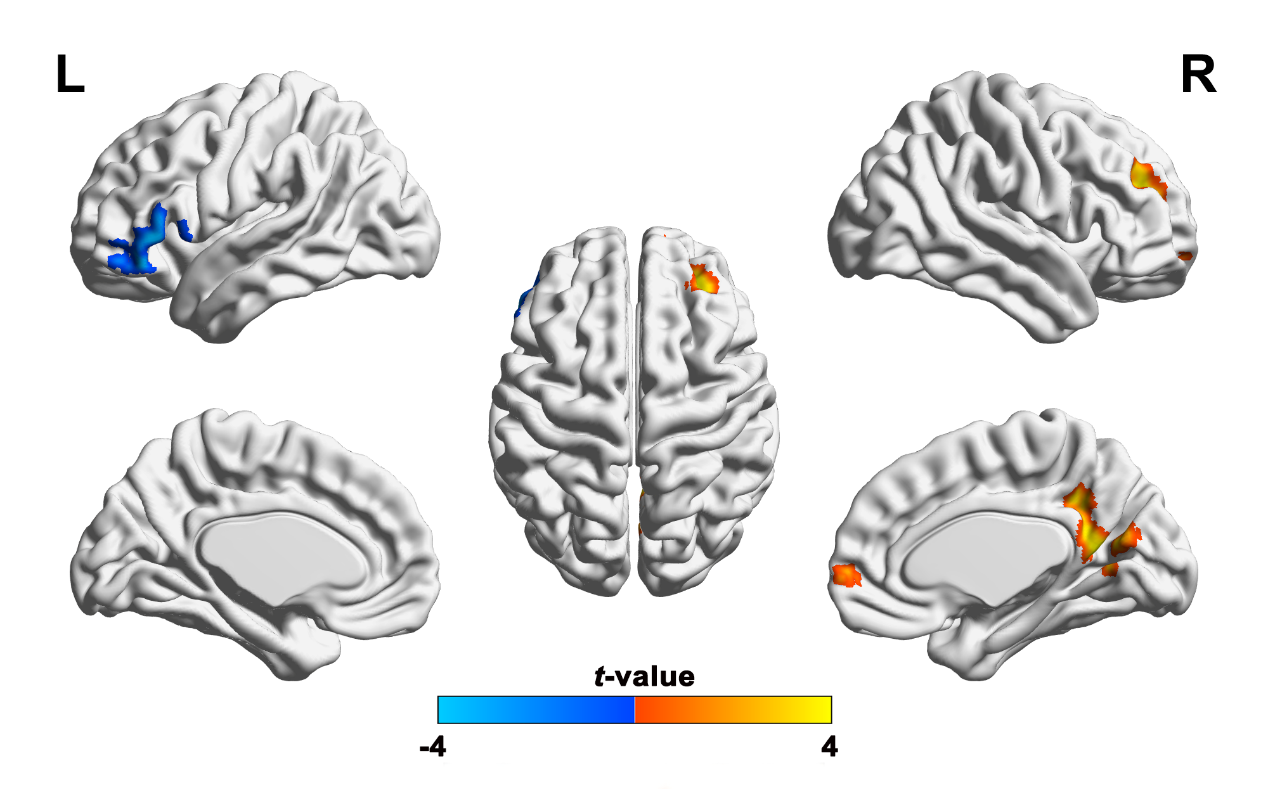
Figure S13 Alterations of CBF/DC ratios in IGE patients compared with HC controlling for the effects of age, sex and education (GRF corrected, P < 0.05), while DC is calculated using a connectivity threshold of 0.25. The warm and cold colors represent significantly increased and decreased CBF/DC ratio in the IGE patients, respectively.


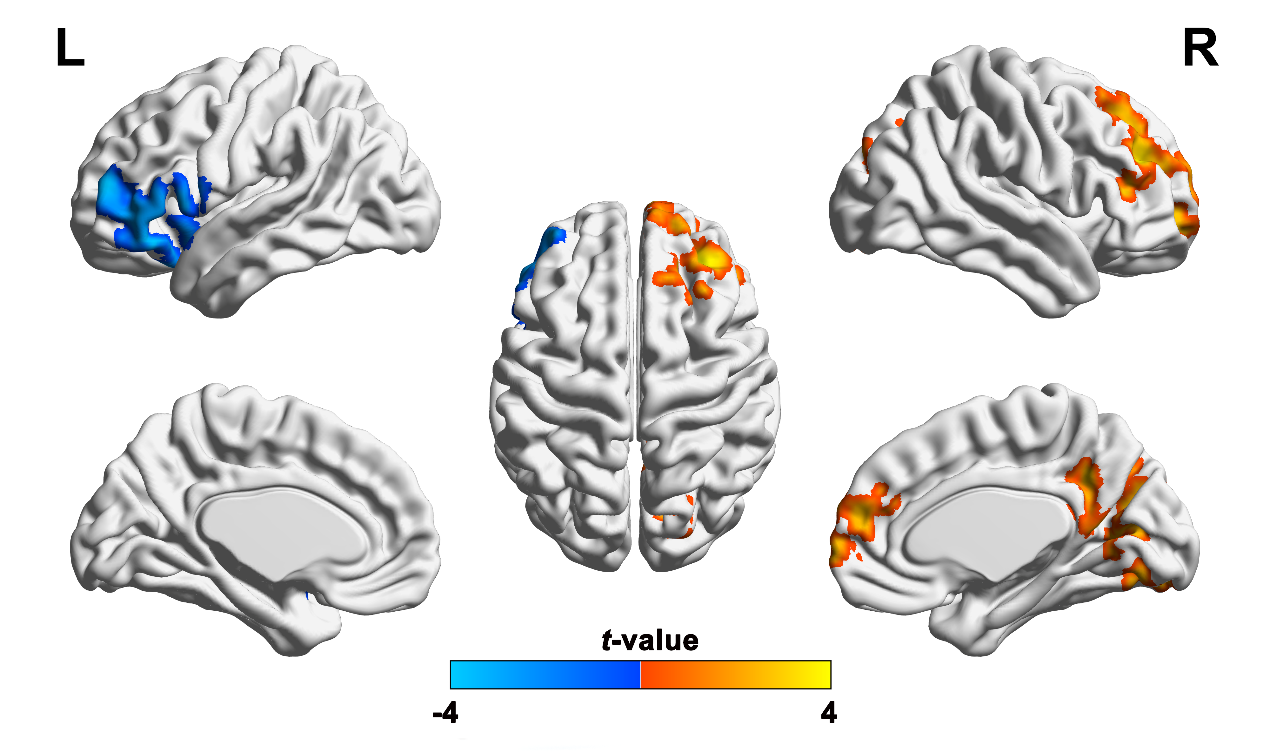
Figure S14 Alterations of CBF/DC ratio in IGE patients compared with HC after correction for medication status and controlling for the effects of age, sex and education (GRF corrected, *P* < 0.05). The warm and cold colors represent significantly increased and decreased CBF/DC ratio in the IGE patients, respectively.


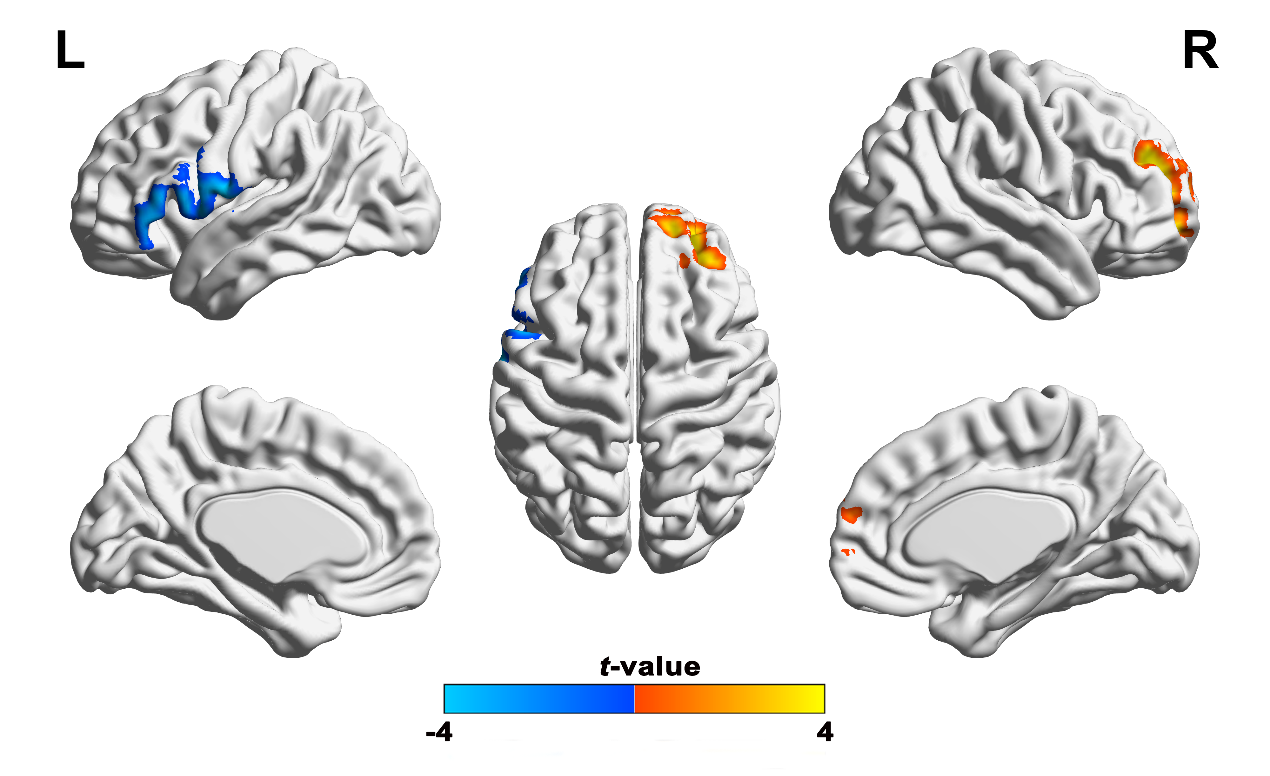


Figure S15 Alterations of CBF/fALFF ratio in IGE patients compared with HC after controlling for the effects of age, sex and education (GRF corrected, *P* < 0.05). The warm and cold colors represent significantly increased and decreased CBF/fALFF ratio in the IGE patients, respectively. The spatial distribution of brain regions with altered CBF/fALFF ratio was similar to that with altered CBF/DC ratio.

We applied a one-step normalization method for normalized the functional images based on the Montreal neurology Institute (MNI) echo-planar-imaging (EPI) template in the paper. However, the average asymmetric brain template for Chinese school-aged children only provided a standard age-specific T_1_-weighted MRI brain template, it did not provide a standard age-specific brain EPI template. Thus, we need to create a standard pediatric EPI template. The process of pediatric EPI template creation consists of three main steps, detailed below. First, the standard T_1_-weighted MNI152 brain template was transformed to standard age-specific T_1_-weighted MRI children brain template (<https://www.nitrc.org/projects/chn-pd>) space by using nonlinear transformation in SPM (<http://www.fil.ion.ucl.ac.uk/spm/>)^1,2^, this registration step was obtained an adult standard MNI brain template to pediatric standard brain template transformation matrix. Then, the transformation matrix from this registration was applied to the EPI template in MNI space, generated a standard pediatric EPI template. Subsequently, the generated pediatric EPI template and the pediatric brain tissue probability maps were used to one-step normalization^3^. Then, the remain preprocessing steps were performed using the same parameters as mentioned in the main text.


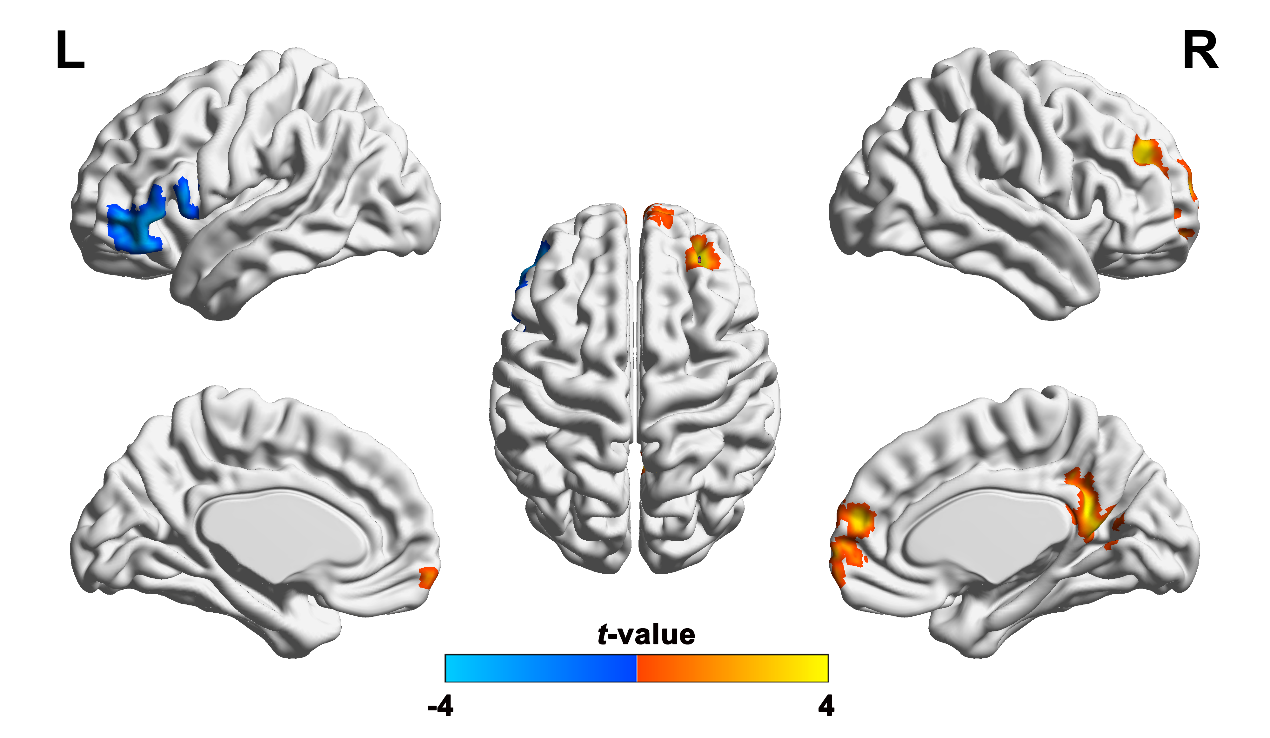


Figure S16 Alterations of CBF/DC ratios in IGE patients compared with HC controlling for the effects of age, sex and education (GRF corrected, P < 0.05), while using pediatric EPI template for spatial normalization. The warm and cold colors represent significantly increased and decreased CBF/DC ratio in the IGE patients, respectively.

**Table S1**

Correlations between CBF, DC and CBF/FCS ratio and clinical variables in IGE patients.

| Brain regions | FIQ | PIQ | VIQ | Duration | Age of onset |
| --- | --- | --- | --- | --- | --- |
| CBF (IGE < HC) |  |  |  |  |  |
| Left inferior frontal gyrus | -0.167(0.416) | -0.117(0.568) | -0.127(0.537) | -0.055(0.788) | 0.070(0.733) |
| CBF (IGE > HC) |  |  |  |  |  |
| Right middle temporal gyrus | 0.161(0.475) | 0.021(0.927) | 0.216(0.334) | -0.109(0.629) | -0.240(0.237) |
| Right superior parietal lobule | 0.247(0.224) | 0.211(0.300) | 0.188(0.357) | 0.040(0.845) | -0.230(0.259) |
| DC (IGE < HC) |  |  |  |  |  |
| Right Posterior Cingulate | -0.304(0.131) | -0.245(0.228) | -0.276(0.172) | 0.364(0.067) | -0.219(0.281) |
| Right Middle Frontal Gyrus | 0.179(0.383) | 0.237(0.244) | 0.072(0.726) | 0.073(0.725) | 0.036(0.860) |
| CBF/DC ratio (IGE < HC) |  |  |  |  |  |
| Left Inferior Frontal Gyrus | 0.006 (0.977) | -0.031(0.977) | 0.082(0.690) | -0.109(0.596) | 0.166(0.419) |
| CBF/DC ratio (IGE > HC) |  |  |  |  |  |
| Right Medial Frontal Gyrus | -0.234(0.250) | **-0.409(0.038)*** | 0.046(0.824) | 0.194(0.324) | 0.023(0.909) |
| Right Posterior Cingulate | 0.268(0.185) | 0.229(0.260) | 0.236(0.245) | -0.229(0.260) | 0.144(0.484) |
| Right Middle Frontal Gyrus | 0.149(0.468) | 0.133(0.516) | 0.170(0.406) | -0.113(0.582) | -0.158(0.442) |

Pearson’s correlation coefficients were used to evaluate correlations between clinical scores and CBF/DC ratios of each significant cluster derived from voxel-wise two sample t-test between the two groups. All values are expressed as the Pearson’s rho (p value). *means significant for *p* < 0.05 and shown in bold; Abbreviations: CBF, cerebral blood flow; DC, Degree centrality; IGE, Idiopathic generalized epilepsy; HC, healthy control; FIQ, full-scale intelligence quotient; PIQ, performance intelligence quotient; VIQ, verbal intelligence quotient; HC, Healthy control.

# References

1. Aubert-Broche B, Fonov VS, García-Lorenzo D, et al. A new method for structural volume analysis of longitudinal brain MRI data and its application in studying the growth trajectories of anatomical brain structures in childhood. *NeuroImage.* 2013;82:393-402.

2. Ashburner J, Friston K. Multimodal image coregistration and partitioning--a unified framework. *Neuroimage.* 1997;6(3):209-217.

3. Zhao T, Liao X, Fonov VS, et al. Unbiased age-specific structural brain atlases for Chinese pediatric population. *Neuroimage.* 2019;189:55-70.
